# A Three-Arm, Randomized, Double-Blind, Placebo-Controlled Study to Evaluate the Safety of *Lactobacillus salivarius* AP-32 and *Bifidobacterium animalis* CP-9 Used Individually in Healthy Infants

**DOI:** 10.3390/nu15153426

**Published:** 2023-08-02

**Authors:** Jui-Fen Chen, Mei-Chen Ou-Yang, Ko-Chiang Hsia, Neonatal Probiotic Use and Safety Research Group, Ching-Min Li, Yao-Tsung Yeh, Hsieh-Hsun Ho

**Affiliations:** 1 Department of Research and Design, Glac Biotech Co., Ltd., Tainan 744, Taiwan; juifen.chen@glac.com.tw (J.-F.C.); shawn.hsia@glac.com.tw (K.-C.H.); chingmin.li@glac.com.tw (C.-M.L.); 2 Department of Pediatrics, Kaohsiung Chang Gung Memorial Hospital, Chang Gung University College of Medicine, Kaohsiung 833, Taiwan; meichenouyang@gmail.com; 3 Chang Gung Memorial Hospital, Taoyuan 333, Taiwan; chchiu@cgmh.org.tw; 4 Aging and Disease Prevention Research Center, Fooyin University, Kaohsiung 831, Taiwan; glycosamine@yahoo.com.tw; 5 Department of Medical Laboratory Science and Biotechnology, Fooyin University, Kaohsiung 831, Taiwan

**Keywords:** probiotics, safety, *Lactobacillus salivarius*, *Bifidobacterium animalis*

## Abstract

Probiotics are considered safe and beneficial to human health. However, the safety of *Lactobacillus salivarius* AP-32 and *Bifidobacterium animalis* CP-9 in infants has not been confirmed. This study was to assess the safety of long-term oral administration of *L. salivarius* AP-32 and *B. animalis* CP-9 in healthy infants compared with placebo. A three-arm, randomized, double-blind, placebo-controlled trial was conducted in healthy, full-term infants. Eighty-eight infants between 7 days and 2 months (60 ± 7 days) of age were selected and randomized to treatment with *L. salivarius* AP-32, *B. animalis* CP-9 or placebo for 4 months. The unblinding indicated subjects were randomized to receive *B. animalis* CP-9 (N = 28), *L. salivarius* AP-32 (N = 29), or placebo (N = 31). A total of 76 infants completed the 4-month treatment with fully compliance. The primary outcome was weight gain, with no significant difference in infant weight at 4 months when comparing AP-32 or CP-9 group with the placebo group, either. The head circumference and recumbent length of the CP-9 group were not significantly different from those of the placebo group. The recumbent length of the AP-32 group was slightly lower than that in the placebo group at month 4, but there was no difference between the two groups in head circumference. Overall, the growth trend of all treatments was similar without significant difference. Furthermore, there were no apparent differences between each group in digestive tolerance, the occurrence of adverse events, crying/fussing time and episodes, alpha diversity, and beta diversity. The CP-9 group showed a significant increase in the abundance of the *Bacteroides* genus, while the AP-32 group demonstrated a significant increase in the abundance of the *Lactobacillus* genus when comparing the two probiotic groups. Our study findings indicate that the oral administration of both AP-32 and CP-9 strains has a positive impact on the maintenance of a healthy gut flora in infants. Long-term use of *L. salivarius* AP-32 or *B. animalis* CP-9 is safe for infants from 7 days to 6 months of age.

## 1. Introduction

Probiotics are defined as “live microorganisms which when administered in adequate amounts confer a health benefit on the host” [1]. Microbes used as probiotics are derived from different genera and species and have been studied for a variety of effects such as promoting gastrointestinal health, preventing infections, and treating diarrhea. Although they have been used safely in foods and dairy products for more than one hundred years, their safety assessment is essential in order to determine the limitations and the population benefiting from general use [2]. Bacterial translocation (i.e., the absorption of live bacteria from the gastrointestinal tract) is an uncommon event that may occur in cases of compromised gastrointestinal integrity, which usually results in bacterial transportation to the mesenteric lymph nodes, liver, spleen, and systemic circulation, possibly resulting in bacteremia, sepsis, and multiple organ failure [3]. Because the digestive system is not fully mature until around six months of age, infants are a population vulnerable to gastrointestinal problems. By passing through rigorous evaluations, certain probiotic organisms have been classified generally recognized as safe (GRAS) for use in food and infant formula by the US Food and Drug Administration (FDA). Pivotal safety data are required to be accessible to the public, and consensus of safety must be agreed by experts under the GRAS notice process [4].

Although the fetal intestine is sterile in the womb, microbial colonization is initiated due to extensive contact in the birth canal during labor [5]. The intestinal microbiota further matures by close contact with the environment and breast milk [6]. Human milk contains several hundred bacterial species, and up to 800,000 bacteria can be ingested by breastfed infants [7]. Being the second integral source of microbes to the infant after the birth canal in vaginally born infants, breast milk-derived microbes make up almost 30% of all bacteria in the infant’s gut [8]. Both *Lactobacillus* and *Bifidobacterium* spp. are found in breast milk and transfer of these microbes to the neonatal gut has been demonstrated using culture- and strain-level discrimination [9,10]. Infant formulas containing *Lactobacillus*, *Bifidobacterium* and/or *Streptococcus thermophilus* are commercially available in parts of Asia, Europe, and in the United States. Numerous studies on probiotics’ use have been conducted on full-term and premature infants, and no short-term or serious events were reported [11,12,13].

*Bifidobacterium animalis* CP-9 was isolated from breast milk, and *Lactobacillus salivarius* AP-32 was isolated from a healthy human gut. Both strains were identified by genome sequencing, and several studies were conducted to investigate their beneficial effects on human health. The safety assessments of *B. animalis* CP-9 were confirmed in rodents [14], and the combination of *B. animalis* CP-9 with other probiotic strains displayed antioxidative activity in middle-aged mice [15]. *L. salivarius* AP-32 displayed antibacterial activity against *Helicobacter pylori*, and reduced inflammatory chemokine expression and lymphocyte infiltration in *H. pylori*-infected rats [16]. In addition, *L. salivarius* AP-32 was able to improve mitochondrial function and alter gut microbiota composition in 6-hydroxydopamin-induced Parkinson’s disease rats [17,18]. Both *B. animalis* CP-9 and *L. salivarius* AP-32 displayed antibacterial activity against oral pathogens, and elevated the IgA concentration in the oral mucosa [19,20]. The combination of *B. animalis* CP-9, *L. salivarius* AP-32, and other probiotic strain (s) was able to reduce inflammation and attenuate glycemic levels in the type 2 diabetic animal model and type 1 diabetic patients [21,22]. Moreover, the multi-strain probiotic supplement containing *B. animalis* CP-9 and *L. salivarius* AP-32 displayed anti-obesity effects in obese rats [23], and reshaped obesity-related gut dysbiosis in obese children [22]. Although *B. animalis* CP-9 and *L. salivarius* AP-32 have had a history of human use for more than one decade, their safe use still needs to be particularly evaluated in infants.

In order to gain a clearer understanding of the safety aspects of probiotic use in infants, we conducted a three-arm trial lasting for 4 months. The subjects were infants aged between 7 days and 2 months, who were randomly divided into three groups: placebo group, *B. animalis* CP-9 group, and *L. salivarius* AP-32 group. We conducted a series of assessments specifically focused on the safety, tolerance, and effects of the probiotic consumption in infants. The primary endpoint was the mean weight gain (change from baseline) at the end of the treatment. Additionally, we observed occurrence of adverse events (AEs), anthropometric measurements, digestive tolerance, incidence of infectious diseases, incidence of allergic diseases, and crying and/or fussing time and episodes. Furthermore, we investigated the modulation of gut microbiota using next-generation sequencing (NGS) analysis.

## 2. Materials and Methods

### 2.1. Study Products

The *Lactobacillus salivarius* AP-32 and *Bifidobacterium animalis* subsp. *lactis* CP-9 were provided by Glac Biotech Co., Ltd. Each capsule of *L. salivarius* AP-32 or *B. animalis* subsp. *lactis* CP-9 contained freeze-dried powder of 2.5 × 10^9^ cfu. Placebo capsules contained 0.5 g maltodextrin without probiotics. All study products were stored in the refrigerator, and the products had been confirmed to be stable for 2 years at this temperature. The study products were packed in blister packs with the same appearance.

### 2.2. Study Design

A randomized, double-blinded controlled study with three study groups was used to investigate the safety of *L. salivarius* AP-32 and *B. animalis* subsp. CP-9 in healthy infants. This research entrusted Efficient Pharma Management Corp. (EffPha, Taipei, Taiwan) to conduct infant admissions by Linkou Chang Gang Memorial Hospital and Kaohsiung Chang Gang Memorial Hospital between September 2019 and August 2022 (registered at ClinicalTrials.gov: NCT04140604). The results of this paper are compiled from the EffPha report.

Infants meeting the following criteria were included in this study. First, informed consent was correctly signed by the parent or legal guardian. Second, the infants were healthy without any medical conditions, full term (≥36 weeks of gestation at birth), and born with a birth weight ≥ 2500 g. Their age was between 7 days and 2 months (60 ± 7 days) at enrollment.

Infants were excluded from the study if they were developmental delayed (weight gain <100 g/week average from birth to the last recorded weight), had major acute or chronic illness (e.g., significant cardiac, respiratory, hematological, gastrointestinal or other systemic diseases, a major developmental or genetic abnormality), cow’s milk protein allergy, history of any allergies to maltodextrin, feeding difficulties, and any use of substances that alter gut microbiota (antibiotics, prebiotics, probiotics, or gastric acid inhibitors) within 2 weeks prior to the study initiation. The breastfed infants were excluded if their mother used antibiotics, prebiotics, or probiotics.

**Figure 1:**
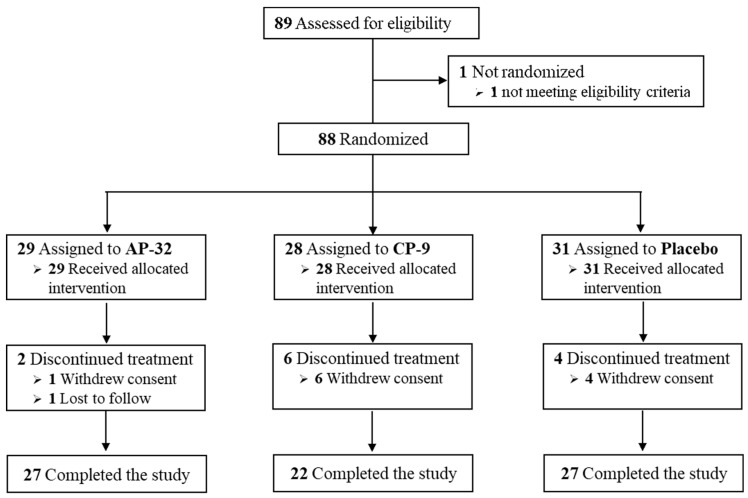
Flow chart of participants.

Sample size was estimated by previous studies, which had mentioned growth as the primary outcome variable of the safety study. Therefore, weight gain was selected as the primary outcome [24]. According to the Scientific Committee for Food Report (SCF/CS/NUT/IF/65), this study was designed to have the power to detect a difference in weight gain equal to 0.5 standard deviations [25]. Assuming the weight gain of the probiotic group was the same as that of the placebo group during the study, 50 evaluable infants per group were required to have 80% power of the test at the significance level of α = 0.05, SD = 0.5. Eighty-eight healthy, full-term infants were randomly assigned into three groups, *L. salivarius* AP-32, *B. animalis* CP-9, and placebo (Figure 1).

In these three different groups, each infant was given the content of one capsule (mixed in infant formula, breast milk, or water) twice daily (morning and evening) for 4 months. The placebo capsules contained maltodextrin, while the probiotic capsules contained *L. salivarius* AP-32 (2.5 × 10^9^ cfu) or *B. animalis* CP-9 (2.5 × 10^9^ cfu), respectively.

### 2.3. Study Outcome and Sample Collection

The primary outcome of this study was average weight gain of infant between baseline and 4 months. Secondary outcomes were occurrence of adverse events (AEs), anthropometric measurements (recumbent length and head circumference), digestive tolerance, incidence of infectious or allergic diseases, and crying and/or fussing time (hours/day) and episodes.

During the study period, infant growth parameters were investigated and recorded at 1, 2, and 4 months. At each visit, the research team reviewed the infant’s daily record of the volume of formula intake/breastfeeding minutes, digestive intolerance symptoms (reflux and gas), sleep duration, frequency of crying/irritability occurrences (hours/day), and episodes. Anthropometric measurements (weight, recumbent length, and head circumference) were also recorded. AEs were assessed based on inquires to the caregivers.

### 2.4. Statistical Analysis

Baseline demographic characteristics of infants such as baseline weight, gender, baseline age category (≤1 or >1 month) were analyzed using linear regression to compare probiotic and placebo groups at a one-sided significance level of 0.05. Analysis of variance (ANOVA) was used to test the differences in the mean values among the three different treatment groups. Anthropometric measurements, crying/fussing time (hours/day), and number of episodes were compared between each group using a two-sample *t*-test. For categorical endpoints such as incidence of AEs and digestive tolerance, an incidence table was used. The rules for interpreting the correlation of r values were provided by Mukaka (2012) [26].

### 2.5. Fecal DNA Extraction

Fecal samples were collected from infants who completed the mITT protocol and were randomly assigned to either the placebo or probiotic treatment group for a duration of 4 months. Total DNA extraction from approximately 200 mg of infant feces was performed using the QIAamp^®^ DNA Mini Kit (QIAGEN Canada, Mississauga, ON, Canada), following the manufacturer’s instructions. The fecal samples were thoroughly suspended in a commercial buffer, and proteinase K was added to facilitate the degradation of proteins. The resulting homogenized solution was subjected to centrifugation, and the supernatant containing the DNA was carefully transferred to a QIAamp Mini spin column. Subsequent washing steps were carried out using an alcohol-containing buffer to remove impurities, and the purified DNA was eluted with a low-salt buffer for further analysis.

### 2.6. Next-Generation Sequencing (NGS) Analysis

Following DNA extraction and purification, the extracted DNA served as the template for PCR amplification. The target region for amplification was the V3-V4 region of the bacterial 16S rRNA gene, using the primer pair 314F (5′-TCGTCGGCAGCGTCAGATGTGTATAAGAGACAGCCTACGGGNGGCWGCAG-3′) and 805R (5′-GTCTCGTGGGCTCGGAGATGTGTATAAGAGACAGGACTACHVGGTATCTAATCC-3′). The amplification was performed with KAPA HiFi HotStart ReadyMix (Roche Sequencing Solutions, Pleasanton, CA, USA [KK2601]), using thermal cycling conditions of 95 °C for 5 min, 30 cycles of 95 °C for 30 s, 60 °C for 30 s, 72 °C for 30 s, and final extension at 72 °C for 5 min. Next, DNA libraries were constructed by ligating Nextera XT Index and Illumina sequencing adapters to the PCR products. The prepared libraries were subjected to paired-end sequencing (2 × 300 bp) on the Illumina MiSeq platform (Illumina, San Diego, CA, USA) by Majorbio Bio-Pharm Technology Co., Ltd. (Shanghai, China), to generate the necessary sequencing data for subsequent analysis.

### 2.7. Bioinformatics Analysis and Statistics

The sequence data underwent quality control and feature table construction using QIIME 2 version 2020.11 (https://qiime2.org, accessed on 23 May 2023) and the DADA2 pipeline [27,28] to address errors and generate accurate results. Subsequently, the reads were merged into amplicon sequence variants (ASVs) for downstream analysis. Alpha diversity, measured by the Shannon index, was used to estimate the diversity of bacterial communities. Beta diversity was assessed using the Bray-Curtis similarity, calculated with MicrobiomeAnalyst (https://www.microbiomeanalyst.ca/, accessed on 29 May 2023) [29,30]. Principal coordinate analysis (PCoA) was performed to visualize changes in species composition across time and space. Taxonomic assignments of the ASVs were based on the Greengenes 13_8 99% OTUs as reference sequences [31]. The data are presented as means ± standard deviation. Statistical comparisons between groups were conducted using Student’s *t*-test. Permutational multivariate analysis of variance (PERMANOVA) was employed to examine statistical differences in beta diversity using QIIME2. A *p*-value less than 0.05 was considered statistically significant.

## 3. Results

### 3.1. Analysis Population

Eighty-eight (88) healthy, full-term infants between 7 days and 2 months (60 ± 7 days) of age were enrolled. They were randomized in a 1:1:1 ratio to receive *L. salivarius* AP-32, *B. animalis* CP-9, or placebo during the 4-month double-blind treatment period. The 88 infants who received at least one dose of the study product were identified as the ITT population. According to the definition of ITT population, there were three other populations defined as follows: (1) modified intent-to-treat (mITT) population included subjects in the ITT population who had at least one post-treatment weight measurement with treatment assignment as randomized; (2) per-protocol (PP) population was subjects in the mITT population with treatment compliance ≥ 80% and without any major protocol deviation (treatment assignment as actual treatment received); (3) safety population consisted of the ITT subjects who had at least one post-treatment safety assessment with treatment assignment as actual treatment received (Table 1).

Moreover, the mITT completers and PP completers were subjects in the mITT or PP population with weight measurements at month 4, respectively. As shown in Table 1, the number of infants in each treatment group was similar across all analysis populations. We used the data from mITT population and mITT completers for further analysis.

Of the 88 infants, 11 (12.5%) withdrew consent, and one (1.1%) was lost to follow-up before completing the treatment. The total number of infants that completed the 4-month treatment was 76 (86.4%), 29 in the AP-32 group, 28 in the CP-9 group, and 31 in the placebo group. The dropout rate in the CP-9 group (21.4%) was slightly higher than in the placebo group (12.9%). A flowchart of participants is shown in Figure 1. Baseline characteristics of mITT population in each group are summarized in Table 2.

There were no significant differences observed among the groups in terms of age, sex, birth weight, and anthropometric data.

### 3.2. Growth of Infants

**Figure 2:**
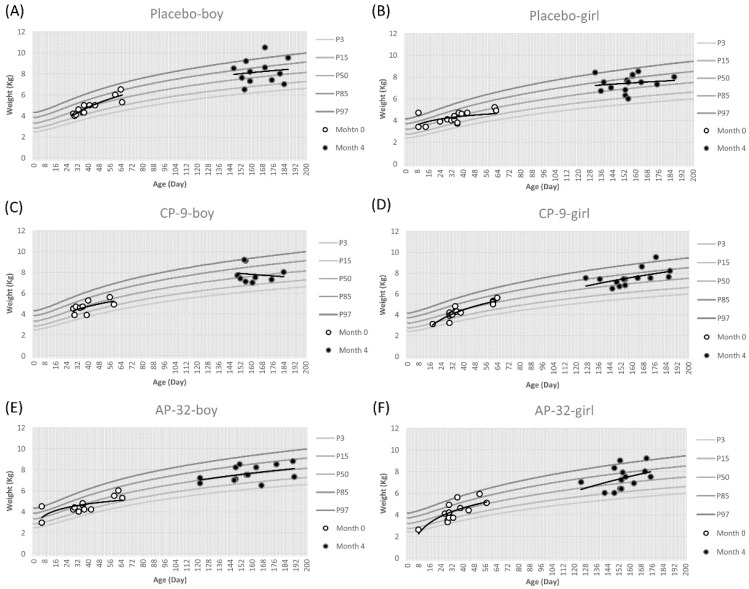
Weight-for-age percentile curves for the mean of boys and girls of three treatment groups between 7 days and 2 months (60 ± 7 days) of age are represented with respect to the standard curves. Placebo group (**A**) boys and (**B**) girls. CP-9 group (**C**) boys and (**D**) girls. AP-32 group (**E**) boys and (**F**) girls. White dots represent the start of the trial at month 0, black dots represent the completion of the trial at month 4. The distribution trend of white dots or black dots is represented by a trend line.

Upon completion of the trial, a comprehensive analysis of the infants’ anthropometric data was performed, focusing on weight, length, and head circumference in relation to both gender and treatment groups. Trend lines were generated for each growth chart to illustrate the trajectory of data points at month 0 and 4. The coefficient “r” was computed to quantify the correlation between the growth data points and the 50th percentile on the WHO growth charts, providing a rigorous assessment of the observed associations. In the placebo group, weight-for-age percentiles for boys ranged from 50% to 85%, while in the *L. salivarius* AP-32 and *B. animalis* CP-9 probiotic groups, weight-for-age percentiles were around 50% (Figure 2).

The average initial weight for boys was approximately 4.7 kg. In the CP-9 group, a moderate positive correlation (r = 0.58) was observed between the weight data points of boys and the 50th percentile on the growth chart (Figure 2C). Conversely, both the placebo and AP-32 groups showed a high positive correlation (placebo: r = 0.83, AP-32: r = 0.71) (Figure 2A,E). By the end of the 4-month period, boys exhibited an average weight gain of up to 7.87 kg. In the AP-32 group, a low positive correlation (r = 0.34) was found between the weight data points of boys and the 50th percentile (Figure 2E), while the placebo and CP-9 groups displayed negligible correlations (placebo: r = 0.11, CP-9: r = 0.08) (Figure 2A,C). Regarding girls, their weight-for-age percentiles ranged from 50% to 85% across all treatment groups (Figure 2). The mean initial weight of girls at the beginning of the study was approximately 4.29 kg. Among the treatment groups, the correlation between the weight data points of girls and the 50th percentile showed a descending order: CP-9, AP-32, and the control groups (CP-9: r = 0.86, AP-32: r = 0.77, placebo: r = 0.64) (Figure 2B,D,F). Over the 4-month period, girls experienced an average weight gain of up to 7.45 kg. The correlation strengths between the weight data points of girls and the 50th percentile differed across the CP-9, AP-32, and placebo groups, ranging from moderate positive (CP-9: r = 0.52, AP-32: r = 0.49) to negligible (placebo: r = 0.12) (Figure 2B,D,F).

**Figure 3:**
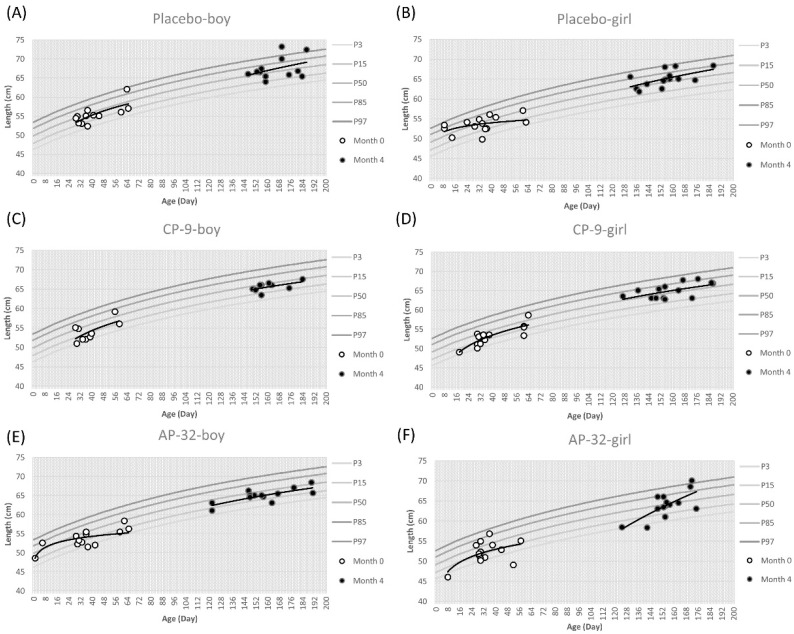
Length-for-age percentile curves for the mean of boys and girls of three treatment groups between 7 days and 2 months (60 ± 7 days) of age are represented with respect to the standard curves. Placebo group (**A**) boys and (**B**) girls. CP-9 group (**C**) boys and (**D**) girls. AP-32 group (**E**) boys and (**F**) girls. White dots represent the start of the trial at month 0, black dots represent the completion of the trial at month 4. The distribution trend of white dots or black dots is represented by a trend line.

Subsequently, we assessed the length-for-age percentiles of boys in the placebo and probiotic groups. The placebo group exhibited length-for-age percentiles ranging from 50% to 85%, while the probiotic groups showed percentiles around 50% (Figure 3).

At the beginning of the study, the average length of boys was approximately 54.32 cm. However, the correlation analysis indicated a negligible correlation between the data points and the 50th percentile across all groups (placebo: r = −0.14, CP-9: r = 0.21, AP-32: r = 0.24) (Figure 3A,C,E). At month 4, boys exhibited an average length gain of 65.98 cm. The strength of correlation between boy length data points and the 50th percentile varied across the AP-32, CP-9, and placebo groups, with correlation coefficients of 0.31, -0.14, and −0.29, respectively (Figure 3A,C,E). In the case of girls, the length-for-age percentiles were approximately 50% in the CP-9 group, while ranging from 50% to 85% in the placebo and AP-32 groups (Figure 3). The average initial length of girls was approximately 52.91 cm. In both the CP-9 and AP-32 probiotic groups, there was a weak negative correlation observed between the girl length data points and the 50th percentile (CP-9: r = −0.33, AP-32: r = −0.30) (Figure 3D,F). In contrast, the placebo group exhibited a negligible correlation (r = 0.17) (Figure 3B). At month 4, girls exhibited an average length gain of up to 64.61 cm. Across all groups, including placebo, CP-9, and AP-32, the correlation between girl length data points and the 50th percentile remained negligible (placebo: r = 0.09, CP-9: r = 0.29, AP-32: r = −0.16) (Figure 3B,D,F).

**Figure 4:**
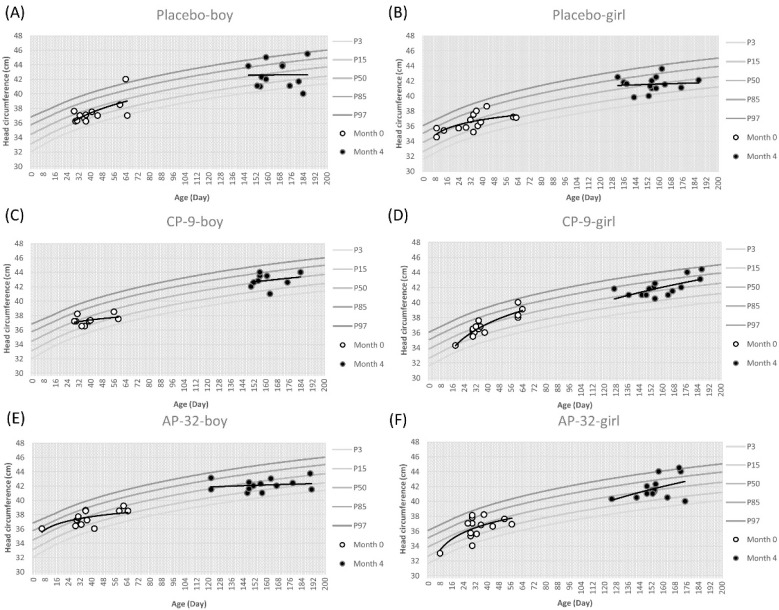
Head circumference-for-age percentile curves for the mean of boys and girls of three treatment groups between 7 days and 2 months (60 ± 7 days) of age are represented with respect to the standard curves. Placebo group (**A**) boys and (**B**) girls. CP-9 group (**C**) boys and (**D**) girls. AP-32 group (**E**) boys and (**F**) girls. White dots represent the start of the trial at month 0, black dots represent the completion of the trial at month 4. The distribution trend of white dots or black dots is represented by a trend line.

In the context of head circumference-for-age percentiles for boys, the placebo and CP-9 groups had approximately 50% percentiles, while the AP-32 group ranged between 15% and 50% (Figure 4).

The average initial head circumference for boys was approximately 37.43 cm, and there was a negligible correlation observed between the data points and the 50th percentile across all groups (placebo: r = −0.20, CP-9: r = 0.13, AP-32: r = 0.24) (Figure 4A,C,E). At month 4, the average head circumference gain for boys reached up to 42.54 cm. When analyzing the correlation between the boy head circumference data points and the 50th percentile, both the CP-9 and AP-32 groups showed negligible correlations (CP-9 group: r = 0.07, AP-32 group: r = −0.03) (Figure 4C,E), while the placebo group exhibited a low negative correlation (r = −0.40) (Figure 4A). The head circumference-for-age percentiles for girls differed across the groups, with the placebo group exhibiting approximately 50% percentiles, while the CP-9 and AP-32 groups ranged between 50% and 85% (Figure 4). The average initial head circumference for girls was approximately 36.60 cm. Correlation analysis revealed a negligible relationship between the girl head circumference data points and the 50th percentile in the placebo and CP-9 groups (placebo: r = 0.13, CP-9: r = −0.21) (Figure 4B,D), while the AP-32 group displayed a low negative correlation (r = −0.31) (Figure 4F). By month 4, the average head circumference gain for girls reached up to 41.76 cm. The data points across all groups exhibited a negligible correlation with the 50th percentile (placebo: r = 0.12, CP-9: r = 0.15, AP-32: r = −0.12) (Figure 4B,D,F).

Next, we further examined the weight, head circumference, and recumbent length of infants at month 4. The mean and mean changes from baseline of weight on month 1 and 2 in mITT population are summarized in Table S1. There were no significant differences in weight at month 4 for infants older than 1 year or ≤1 year of age, or gender, between the two probiotic groups and the placebo group (*p* > 0.05) (Table 3).

No significant differences were observed in head circumference or recumbent length between the CP-9 and the placebo group (Table 4).

The recumbent length of the AP-32 group was slightly lower than that of the placebo group at month 4, but there was no difference between the two groups in head circumference. Moreover, there was no significant difference between the probiotic and placebo groups in the crying/fussing time and episodes at month 4 (*p* > 0.05) (Table 5).

### 3.3. Infant Health

Infants who experienced at least one infectious or allergic disease during the study were reported as having an adverse event (AE), and the results are summarized in Table 6.

A total of 12 infants had infectious diseases during the trial, including 5 in the AP-32 group, and 2 each in the CP-9 group and placebo group. The results also revealed that a common infectious disease was upper respiratory disease, with one infant in each group affected. Three infants in each of the three treatment groups had allergic diseases. The most frequently reported allergic disease was atopic dermatitis, which was reported in 4 infants—1 each in the AP-32 and placebo groups and 2 in the CP-9 group. None of these AEs were considered study product-related. Notably, 86% of infants had no AEs during the study. The frequency of AEs occurred randomly among the three treatment groups, and there was no causal relationship between AEs and study products. Most infants showed no changes in the symptoms of digestive tolerance during the study period (Table S2). There appeared to be no differences in the severity of regurgitation or frequency of flatulence between the placebo group and the AP-32 or CP-9 groups.

### 3.4. Gut Microbiota Modulation

**Figure 5:**
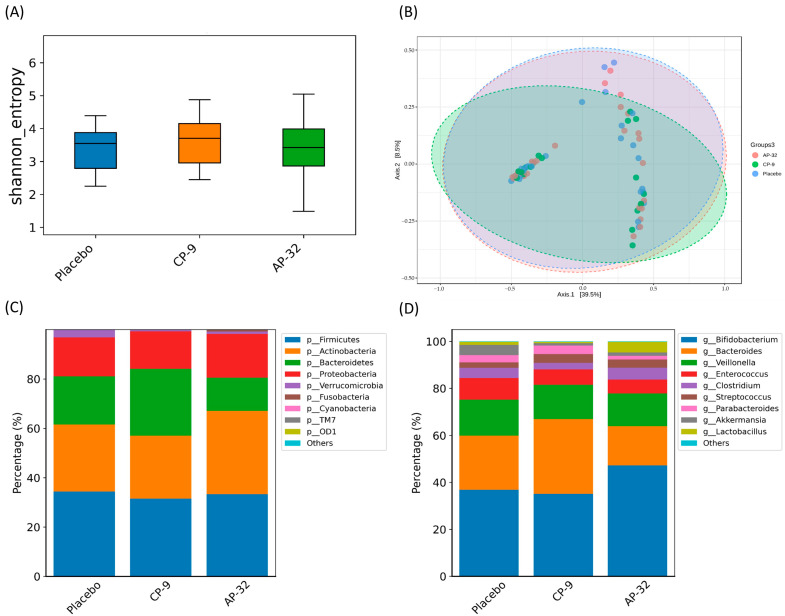
Effects of oral administration of placebo, CP-9, and AP-32 on the modification of infant gut microbiome after 4 months. (**A**) Alpha diversity. (**B**) Beta diversity. (**C**) Top 10 most abundant bacterial phylum. (**D**) Top 10 most abundant bacterial genera. Parents of one infant in the placebo group did not provide a stool sample. Another infant in the CP-9 group was excluded for not belonging to the mITT completers. These two infants were excluded from gut microbiota analysis.

To investigate the influence of probiotic supplementation on infant gut microbiota composition, we performed DNA extraction from fecal samples obtained from all study groups at month 4. The extracted DNA was subjected to thorough analysis using next-generation sequencing methods to provide detailed insights into the microbial community. Alpha diversity and beta diversity indexes were utilized to evaluate the species richness and evenness within the same group and between different groups, respectively. Notably, there were no statistically significant differences in either alpha or beta diversity observed between the placebo and probiotic groups (Figure 5A,B).

The infant gut microbiota in all study groups consisted predominantly of four dominant bacterial phyla: Firmicutes, Actinobacteria, Bacteroidetes, and Proteobacteria, which collectively represented approximately 90% of the total microbial community (Figure 5C). A statistically significant elevation in the relative abundance of Bacteroidetes at the phylum level was noted in the CP-9 group in comparison to the AP-32 group (*p* = 0.032) (Figure 5C). Significantly, the primary genera—*Bifidobacterium*, *Bacteroides*, *Veillonella,* and *Enterococcus*—comprised more than 80% of the infant gut microbiota across all study groups (Figure 5D). The CP-9 group exhibited a significant increase in the relative abundance of *Bacteroides* at the genus level compared to the AP-32 group (*p* = 0.062) (Figure 5D). Conversely, the AP-32 group demonstrated a significantly higher relative abundance of *Lactobacillus* compared to the CP-9 group (*p* = 0.042) (Figure 5D). Furthermore, no significant differences were found in the relative abundance of the top 10 phyla or genera across the different groups (Figure 5C,D).

## 4. Discussion

*Lactobacillus* and *Bifidobacterium* are the bacterial genera most commonly used as probiotics [32]. *B. animalis* and *L. salivarius* have been used as functional food for decades, which also means that AP-32 and CP-9 are safe to eat for the general population. In this study, we evaluated the safety of AP-32 and CP-9 in healthy infants between 7 days and 2 months of age. First, anthropometric measurements such as weight, length, and head circumference in three groups were mostly located at the 50th percentile at month 0. These values were more scattered on the growth charts at month 4, but the trend line for anthropometric data was still around the 50th percentile across all treatment groups. One of the reasons for the scattered data may be that the infants in this trial had different growth rates at different ages. This result was consistent with the growth curve of infants, and the distribution area of the percentile curve from the 3rd to 97th gradually increased with age. For all groups, we observed a decreasing trend in the strength of the positive correlation between age and weight, length, and head circumference from 0 to 4 months. The infant growth curve results revealed no significant differences in infants’ increasing weight, length, and head circumference between the placebo and probiotic groups (Figure 2, Figure 3 and Figure 4).

The growth of infants is a complex process. Many factors are known to affect an infant’s growth, including genes and environment [33]. Birth weight is known to be one of the important indicators of infant nutrition absorption and genetics [34,35]. The birth weight of infants in different groups was between 2500 g and 4650 g, which suggested that there were individual differences in nutrient absorption capacity (Table 2). Different sources of nutrition, such as formula milk or breast milk, may influence the growth of the infant. Lind et al. indicated that breastfed infants gain more weight, length, and BMI during the first 2–3 months of life, and their growth rates slowed down until 12 months [36]. In addition, some studies indicate that infant growth is affected by the mother’s education [33,37]. Well-educated women are more likely to have high-paying jobs and long-term relationships, which may affect the health and survival of children [38]. Whereas slight differences were found in the anthropometric data among three groups, the overall growth trend was very similar between the two probiotic groups and the placebo group.

There was no significant difference between the CP-9 and placebo groups in the data of infant weight, length, and head circumference at month 4 (Table 3 and Table 4). The recumbent length of the AP-32 group was slightly lower than that of the placebo group at the end of the trial (Table 4). Nevertheless, there was no difference between the two groups in weight and head circumference (Table 3 and Table 4). Probiotics are commonly used to maintain healthy gut flora in children. Onubi et al. observed no significant effect of probiotics on child growth in developed country studies, but it had a positive effect on undernourished and healthy children in developing countries [39]. Catania et al. assessed 79 studies suggesting that probiotics may have a small effect on weight and height in children from low- and middle-income countries, but not in children from high-income countries. Furthermore, there was no evidence that probiotics increased the risk of AEs [40]. These studies suggested that the economic inequality was one of the factors affecting children’s growth. The intervention of probiotics may not have a significant impact on the growth of children.

One limitation of this study was the insufficient sample size. The *p* values are only given to demonstrate the strength of evidence and should be interpreted with caution. Thiese et al. recommended that *p* values should be considered as a continuous spectrum rather than a criterion of significance or not [41]. Factors such as sample size, bias, and random error may affect the *p* values. Therefore, we should be more cautious about the *p* values when forming conclusions. For sample size determination, we had expected 50 evaluable infants per group would be required to have 80% power of the test at the significance level of alpha = 0.05, SD = 0.5. Due to the COVID-19 pandemic, the study was terminated early, with about 30 subjects in each arm. Hence, the nomogram was used to calculate that when the sample size was about 30, the standard deviation of the trial was about 0.7 (power = 80%, alpha = 0.05) [42,43], although our standard deviation was slightly higher than 0.5, indicating that some values were more widely spread out from the mean. Similar growth trends were found in infant anthropometric data in the placebo group and the AP-32 and CP-9 groups.

Colic syndrome is commonly observed in infants younger than 4 months. Chen et al. revealed that oral administration of probiotics could effectively reduce the episodes and time of infant crying caused by colic [44]. In our study, the average daily crying/fussing episodes in each group was 2.4 in AP-32, 2.0 in CP-9, and 1.9 in placebo (Table 5). The average crying/fussing time in each group was 25.7 min/d in AP-32, 18.9 min/d in CP-9, and 19.4 min/d in placebo. These results demonstrated no apparent difference in crying/fussing time and episodes when comparing the AP-32 or CP-9 group with the placebo group, either.

Regarding the health status of infants, there were no significant differences in infectious and allergic diseases among the three groups. Of these, a minority of infants in all groups had a history of upper respiratory infection or atopic dermatitis (Table 6). Acute respiratory infection is a common disease in children; it is also one of the leading causes of child mortality [45]. Oral administration of *Lactobacillus* can be beneficial in respiratory health via the gut-lung axis [46]. The efficacy of *Lactobacillus* on the respiratory tract is strain-dependent, and the benefits may vary among *Lactobacillus* species. Previous studies also suggested the beneficial effects of *Bifidobacterium* on reducing respiratory infections [47,48,49]. However, the efficacy of probiotics in treating atopic dermatitis is still unproven, and it is recommended to accumulate more experimental data before interpretation [50,51]. No association of probiotics with infection or allergic diseases was found in this study. AEs occurred equally among the three treatment groups, showing no association of AEs with the probiotic intervention.

A significant disparity was observed at the phylum level, with the CP-9 group displaying a markedly higher relative abundance of Bacteroidetes compared to the AP-32 group (Figure 5C). Firmicutes and Bacteroidetes are the two primary bacterial phyla that dominate the human gut, constituting more than 90% of the total microbial community [52]. The Firmicutes-to-Bacteroidetes (F/B) ratio is a recognized indicator used to assess the efficiency of nutrient absorption in the human intestine. An increased F/B ratio and modulation of the gut microbiota are thought to reflect an enhanced ability to ferment dietary polysaccharides into short-chain fatty acids (SCFAs) [53]. The Firmicutes phylum comprises well-known producers of SCFAs, such as *Anaerostipes* spp., *Coprococcus catus*, *Eubacterium rectale*, *Eubacterium hallii*, *Faecalibacterium prausnitzii,* and *Roseburia* spp [53,54]. Additionally, SCFAs are estimated to contribute approximately 10% of the total daily dietary energy supply in humans [53]. The findings suggest a positive association between efficient nutrient absorption and a higher abundance of specialized energy-harvesting bacterial species in the gut microbiota. Specifically, the AP-32 group displayed a significantly lower relative abundance of Bacteroidetes compared to the CP-9 group, resulting in a higher F/B ratio in the AP-32 group. These results provide further support for the hypothesis that supplementation with AP-32 has the potential to improve nutrient utilization in infants.

In addition, we observed a significant increase in the relative abundance of the genus *Bacteroides* in the CP-9 group (Figure 5D). Previous studies have provided empirical support for a symbiotic relationship between *Bacteroides* and *Bifidobacterium*, as *Bacteroides* species possess the enzymatic capacity to metabolize a diverse range of polysaccharides, thereby facilitating their utilization by *Bifidobacterium* [55,56,57,58]. *Bacteroides* species exhibiting xylanolytic activity have been demonstrated to stimulate the proliferation of *B. animalis* subsp. *lactis* during co-culture fermentation [55]. Moreover, the enzymatic hydrolysis of wheat arabinoxylan (WAX) or birchwood glucuronoxylan (BGX) by *Bacteroides ovatus* has been shown to promote the growth of *B. adolescentis* [57,59]. The supplementation of *Bifidobacterium* CP-9 appears to be positively correlated with an increase in *Bacteroides* abundance. Significantly, the AP-32-treated group exhibited a higher relative abundance of *Lactobacillus* in the infant gut microbiota (Figure 5D). The oral administration of AP-32, a member of the *Lactobacillus* genus, effectively increased the presence of *Lactobacillus* in the intestinal tract of infants. These outcomes collectively indicated that the oral administration of AP-32 or CP-9 confers beneficial effects on the establishment and maintenance of a healthy gut microbiota in infants.

## 5. Conclusions

There were no significant differences between placebo and probiotic groups in anthropometric measures, digestive tolerance, AEs, crying/fussing time and episodes, alpha diversity, and beta diversity. The higher F/B ratio in the AP-32 group indicated potential enhanced nutrient utilization in the infant gut. Additionally, the CP-9 group exhibited a significant increase in the relative abundance of *Bacteroides*, suggesting a symbiotic association with *Bifidobacterium*. In contrast, the AP-32 group showed a significant increase in the relative abundance of *Lactobacillus*. In conclusion, the findings from this study suggest that the daily long-term oral administration of *L. salivarius* AP-32 or *B. animalis* CP-9 in infants aged 7 days to 6 months is safe, as no safety concerns were identified. Further research is warranted to investigate the potential health effects of these probiotics, either individually or in combination, particularly in the context of infants and children.

## Figures and Tables

**Table 1:** Analysis populations.

Analysis Population	Total	AP-32	CP-9	Placebo
Intent-to-treat (ITT)	88	29	28	31
Modified intent-to-treat (mITT)	86 (97.7%)	29 (100.0%)	26 (92.9%)	31 (100.0%)
Per-protocol (PP)	68 (77.3%)	24 (82.8%)	20 (71.4%)	24 (77.4%)
Safety	86 (97.7%)	29 (100.0%)	26 (92.9%)	31 (100.0%)
mITT completers	75 * (85.2%)	27 (93.1%)	21 (75.0%)	27 (87.1%)
PP completers	61 (69.3%)	23 (79.3%)	16 (57.1%)	22 (71.0%)

* A total of 76 infants completed the 4-month study (Figure 1); however, one infant in the CP-9 group had her measurement outside the analysis window (98 ≤ [assessment date at month 4 − date of the first dosing] ≤ 142) and was excluded from the mITT completer population.

**Table 2:** Demographics and baseline characteristics (mITT population).

	Total N = 86	AP-32 N = 29	CP-9 N = 26	Placebo N = 31
Age (month) ^a^				
Mean (SD)	1.3 (0.5)	1.2 (0.5)	1.3 (0.4)	1.3 (0.5)
Median	1.2	1.1	1.2	1.2
Min, max	0, 2	0, 2	1, 2	0, 2
Gender				
Male	37 (43.0%)	14 (48.3%)	10 (38.5%)	13 (41.9%)
Female	49 (57.0%)	15 (51.7%)	16 (61.5%)	18 (58.1%)
Gestational age (week) ^b^				
Mean (SD)	38.5 (1.0)	38.5 (1.0)	38.5 (0.9)	38.6 (1.0)
Median	38.5	38.1	38.7	38.4
Min, max	36, 41	36, 40	37, 40	37, 41
Delivery method				
Natural birth	57 (66.3%)	17 (58.6%)	19 (73.1%)	21 (67.7%)
C-section	29 (33.7%)	12 (41.4%)	7 (26.9%)	10 (32.3%)
Weight at birth (g)				
Mean (SD)	3157.1 (419.9)	3182.2 (519.6)	3154.6 (374.2)	3135.6 (360.2)
Median	3140.0	3100.0	3170.0	3160.0
Min, max	2500, 4650	2500, 4650	2580, 4290	2520, 4480
Recumbent length at birth (cm)				
Mean (SD)	50.2 (2.1)	49.7 (2.0)	50.7 (2.0)	50.2 (2.1)
Median	50.0	49.5	50.5	50.0
Min, max	45, 57	45, 56	45, 54	46, 57
Head circumference at birth (cm)				
Mean (SD)	33.8 (1.3)	33.8 (1.5)	33.8 (1.1)	33.8 (1.3)
Median	34.0	33.5	34.0	34.0
Min, max	31, 36	31, 36	31, 36	31, 36
Weight at baseline ^c^ (g)				
Mean (SD)	4409.8 (733.5)	4387.6 (811.0)	4411.5 (676.7)	4429.0 (726.7)
Median	4300.0	4200.0	4300.0	4300.0
Min, Max	2600, 6500	2600, 6000	3100, 5600	3100, 6500
Recumbent length at baseline ^c^ (cm)				
Mean (SD)	53.4 (2.5)	52.8 (2.6)	53.3 (2.4)	54.0 (2.6)
Median	53.4	52.7	53.4	53.8
Min, max	46, 62	46, 58	49, 59	48, 62
Head circumference at baseline ^c^ (cm)				
Mean (SD)	36.9 (1.4)	37.0 (1.4)	37.0 (1.2)	36.7 (1.5)
Median	36.8	37.0	36.8	36.6
Min, max	33, 42	33, 39	34, 40	33, 42

^a^ Age (month) = (date of visit 1 − date of birth)/30. ^b^ Gestational age (week): number of days was converted to the number of weeks by dividing the value (in days) by 7. ^c^ Baseline: the last non-missing measurement before the first dosing of study products.

**Table 3:** Summary of mean and mean changes from baseline of weight at month 4 in mITT completers.

			Weight Gain at Month 4 (g)			
Age (Month)	Gender	Statistics	Total	AP-32	CP-9	Placebo
≤1	Male	n	6	3	1	2
		Mean (SD)	3226.7 (685.5)	3120.0 (563.2)	3200.0 (-)	3400.0 (1272.8)
		Median	3050.0	2900.0	3200.0	3400.0
		Min, max	2500, 4300	2700, 3760	3200, 3200	2500, 4300
	Female	n	12	4	2	6
		Mean (SD)	3450.0 (702.6)	3375.0 (950.0)	3300.0 (1414.2)	3550.0 (345.0)
		Median	3550.0	3400.0	3300.0	3550.0
		Min, max	2300, 4400	2300, 4400	2300, 4300	3100, 4100
>1	Male	n	28	10	8	10
		Mean (SD)	3160.7 (845.2)	3030.0 (600.1)	3125.0 (799.6)	3320.0 (1112.4)
		Median	3100.0	3000.0	3100.0	3150.0
		Min, max	1700, 5500	2000, 4100	1700, 4400	1700, 5500
	Female	n	29	10	10	9
		Mean (SD)	3075.9 (681.7)	3010.0 (772.4)	3250.0 (696.4)	2955.6 (591.8)
		Median	3100.0	3150.0	3200.0	2800.0
		Min, max	1900, 4600	1900, 4600	2200, 4300	2200, 3800

**Table 4:** Recumbent length and head circumference at month 4 (mITT completers).

Statistics	AP-32 N = 27	CP-9 N = 21	Placebo N = 27
Recumbent length (cm)			
Mean (SD)	64.4 (2.7)	65.2 (1.7)	66.1 (2.7)
Median	64.6	65.2	65.7
Min, max	58, 70	63, 68	62, 73
*p*-value	0.0210	0.1640	1.0000
Head circumference (cm)			
Mean (SD)	41.9 (1.2)	42.3 (1.2)	42.0 (1.5)
Median	41.6	42.0	41.8
Min, max	40, 45	41, 44	40, 46
*p*-value	0.7524	0.4920	1.0000

**Table 5:** Comparison of crying/fussing episodes and time at month 4 (mITT completers).

Statistics	AP-32 N = 27	CP-9 N = 21	Placebo N = 26 *
Crying/fussing episodes (episodes/day)			
Mean (SD)	2.4 (1.7)	2.0 (1.7)	1.9 (1.6)
Median	2.3	1.7	1.8
Min, max	0, 6	0, 6	0, 5
Crying/fussing time (minutes/day)			
Mean (SD)	25.7 (24.4)	18.9 (17.9)	19.4 (23.0)
Median	15.0	11.7	10.8
Min, max	0, 90	0, 55	0, 88

* One infant in the placebo group had weight measurement but not crying/fussing record at month 4.

**Table 6:** Summary of infectious diseases and allergic diseases (mITT population).

	Total N = 86	AP-32 N = 29	CP-9 N = 26	Placebo N = 31
Infectious diseases	12 (14.0%)	5 (17.2%)	2 (7.7%)	5 (16.1%)
Acarodermatitis	1 (1.2%)	0	0	1 (3.2%)
Bronchiolitis	1 (1.2%)	0	1 (3.8%)	0
Corona virus infection	1 (1.2%)	1 (3.4%)	0	0
Enteritis	1 (1.2%)	0	0	1 (3.2%)
Nasopharyngitis	2 (2.3%)	0	0	2 (6.5%)
Oral candidiasis	1 (1.2%)	1 (3.4%)	0	0
Upper respiratory tract infection	3 (3.5%)	1 (3.4%)	1 (3.8%)	1 (3.2%)
Urinary tract infection	2 (2.3%)	2 (6.8%)	0	0
Allergic diseases	9 (10.5%)	3 (10.3%)	3 (11.5%)	3 (9.6%)
Rhinitis allergic	1 (1.2%)	1 (3.4%)	0	0
Dermatitis atopic	4 (4.7%)	1 (3.4%)	2 (7.7%)	1 (3.2%)
Dermatitis contact	3 (3.5%)	1 (3.4%)	1 (3.8%)	1 (3.2%)
Infantile eczema	1 (1.2%)	0	0	1 (3.2%)

## Data Availability

The datasets used and/or analyzed during current study are available from the corresponding authors on request.

## Supplementary Materials

The following supporting information can be downloaded at: https://www.mdpi.com/article/10.3390/nu15153426/s1, Table S1: Summary of mean and mean changes from baseline of weight at month 1 and 2 in mITT population; Table S2: Shift table of digestive tolerance (mITT population).
