# Supplementary material for: A Three-Arm, Randomized, Double-Blind, Placebo-Controlled Study to Evaluate the Safety of Lactobacillus salivarius AP-32 and Bifidobacterium animalis CP-9 Used Individually in Healthy Infants
Source: Nutrients. 2023 Aug 2;15(15):3426. doi: 10.3390/nu15153426 (PMC10421338; doi:10.3390/nu15153426)
Supplement: Supplementary file 1 [file nutrients-15-03426-s001.zip › nutrients-2526500-supplementary.pdf]

Supplementary data

**Table S1.** Summary of mean and mean changes from baseline of weight on month 1 and 2 in mITT population

| **Weight (g)** | | **Actual Value** | | | | | | **Changes from Baseline** | | | | | |
|---|---|---|---|---|---|---|---|---|---|---|---|---|---|
| **Study Arm** | **Analysis Visit*** | **N** | **Mean** | **SD** | **Median** | **Min** | **Max** | **N** | **Mean** | **SD** | **Median** | **Min** | **Max** |
| AP-32 | Month 1 | 28 | 5403.6 | 790.9 | 5250.0 | 4100 | 7800 | 28 | 1034.3 | 445.6 | 1050.0 | -200 | 1900 |
| | Month 2 | 28 | 6246.4 | 835.1 | 6150.0 | 5000 | 8300 | 28 | 1852.1 | 562.8 | 1700.0 | 900 | 3000 |
| CP-9 | Month 1 | 23 | 5369.6 | 807.6 | 5200.0 | 4200 | 7100 | 23 | 987.0 | 419.2 | 900.0 | 200 | 1800 |
| | Month 2 | 24 | 6275.0 | 969.6 | 6000.0 | 4500 | 8200 | 24 | 1816.7 | 615.5 | 1850.0 | 700 | 3000 |
| | 155 | 1 | 7600.0 | - | - | - | - | 1 | 3600.0 | - | - | - | - |
| Placebo | Month 1 | 31 | 5561.3 | 808.6 | 5700.0 | 4200 | 7000 | 31 | 1132.3 | 385.9 | 1100.0 | 500 | 1800 |
| | Month 2 | 28 | 6432.1 | 851.2 | 6500.0 | 5100 | 8200 | 28 | 1935.7 | 510.8 | 2000.0 | 1000 | 3200 |

**Table S2.** Shift table of digestive tolerance (mITT population)

| | | AP-32 N=29# | CP-9 N=26# | Placebo N=31# |
|---|---|---|---|---|
| Regurgitation | | | | |
| Baseline* | Post-baseline** | n (%) | n (%) | n (%) |
| None | None | 1 (3.4%) | 3 (11.5%) | 2 (6.5%) |
| | Mild | 4 (13.8%) | 0 | 3 (9.7%) |
| | Moderate | 1 (3.4%) | 0 | 0 |
| | Severe | 1 (3.4%) | 0 | 1 (3.2%) |
| Mild | None | 0 | 0 | 2 (6.5%) |
| | Mild | 15 (51.7%) | 14 (53.8%) | 11 (35.5%) |
| | Moderate | 2 (6.9%) | 2 (7.7%) | 4 (12.9%) |
| | Severe | 2 (6.9%) | 0 | 1 (3.2%) |
| Moderate | None | 0 | 0 | 1 (3.2%) |
| | Mild | 0 | 2 (7.7%) | 2 (6.5%) |
| | Moderate | 1 (3.4%) | 0 | 1 (3.2%) |
| | Severe | 1 (3.4%) | 0 | 0 |
| Severe | None | 0 | 0 | 0 |
| | Mild | 1 (3.4%) | 3 (11.5%) | 1 (3.2%) |
| | Moderate | 0 | 0 | 2 (6.5%) |
| | Severe | 0 | 0 | 0 |
| Flatulence | | | | |
| Baseline* | Post-baseline** | n (%) | n (%) | n (%) |
| None | None | 2 (6.9%) | 3 (11.5%) | 3 (9.7%) |
| | Seldom | 4 (13.8%) | 5 (19.2%) | 5 (16.1%) |
| | Often | 0 | 0 | 1 (3.2%) |
| Seldom | None | 2 (6.9%) | 2 (7.7%) | 0 |
| | Seldom | 13 (44.8%) | 8 (30.8%) | 15 (48.4%) |
| | Often | 2 (6.9%) | 2 (7.7%) | 1 (3.2%) |
| Often | None | 0 | 0 | 1 (3.2%) |
| | Seldom | 3 (10.3%) | 1 (3.8%) | 3 (9.7%) |
| | Often | 3 (10.3%) | 3 (11.5%) | 2 (6.5%) |

# Number of evaluable infants

* Baseline: the highest severity before the first dosing of study products per subject

** Post-baseline: the highest severity after baseline per subject

Two infant in the CP-9 group had baseline records but missing post-baseline data.
